# Comparative dynamics of peritoneal cell immunophenotypes in sheep during the early and late stages of the infection with *Fasciola hepatica* by flow cytometric analysis

**DOI:** 10.1186/s13071-018-3250-5

**Published:** 2018-12-14

**Authors:** Raúl Pérez-Caballero, F. Javier Martínez-Moreno, Rafael Zafra, Verónica Molina-Hernández, Isabel L. Pacheco, M. Teresa Ruiz-Campillo, Alejandro Escamilla, José Pérez, Álvaro Martínez-Moreno, Leandro Buffoni

**Affiliations:** 1 Animal Health Department (Parasitology and Parasitic Diseases), Faculty of Veterinary Medicine, University of Córdoba, Campus de Rabanales, Ctra. Madrid-Cádiz, km 396, 14014 Córdoba, Spain; 2 Anatomy and Comparative Pathology Department, Faculty of Veterinary Medicine, University of Córdoba, Campus de Rabanales, Ctra. Madrid-Cádiz, km 396, 14014 Córdoba, Spain

**Keywords:** Flow cytometry, NEJ, *Fasciola hepatica*, Peritoneal cells, Recruitment

## Abstract

**Background:**

The peritoneal cell populations (PCP) are thought to play a crucial role during the early immune response in *Fasciola hepatica* infection while newly excysted juveniles (NEJ) are migrating in the peritoneal cavity (PC) towards the liver. In this study, we aimed to determine the immunophenotypes of the PCP and to analyse the dynamics of the recruitment of the PCP during the early and late stage of the infection in sheep infected with *F. hepatica*.

**Methods:**

Thirty-seven sheep were divided into three groups: Group 1 (*n* = 20) and 2 (*n* = 10) were challenged with *F. hepatica*, Group 3 (*n* = 7) was not infected and remained as uninfected control (UC). After the slaughtering, peritoneal lavages were carried out to isolate peritoneal cell populations at 1, 3, 9 and 18 days post-infection (dpi) for Group 1 and at 14 weeks post-infection (wpi) for Group 2 and 3. Flow cytometry was conducted to assess the dynamics of peritoneal cavity cell populations.

**Results:**

TCD4 cells showed a significant decrease at 1 and 18 dpi when compared to UC; no statistical differences were detected for TCD8 and WC1^+^γδ during the early stage of the infection with respect to the UC. CD14 cells exhibited a decreasing trend, with a significant decrease at 9 and 18 dpi when compared to the UC. The dynamics of MHCII and CD83 cells showed a similar increasing pattern from 3 to 18 dpi. During the chronic stage, both TCD4 and TCD8 cells showed no significant differences when compared to the UC, although a slight but statistically significant higher level of WC1^+^γδ cells was observed. A lower percentage of antigen-presenting cells (APCs) was detected with respect to the UC.

**Conclusions:**

The recruitment of the lymphocytes subsets did not show a significant increase during the course of the infection and only WC1^+^γδ cells displayed a significant increase at the chronic stage. For the CD14, a decreasing trend was observed during the early stage, which was statistically significant at the chronic stage of the infection. Peritoneal CD83 and MHCII cells developed an increasing trend during the early stage of infection, and showed a significant decrease at the late stage of the infection.

**Electronic supplementary material:**

The online version of this article (10.1186/s13071-018-3250-5) contains supplementary material, which is available to authorized users.

## Background

*Fasciola hepatica* is a globally spread highly pathogenic trematode which mainly occurs in domestic ruminants as a chronic disease and produces major economic losses in terms of production loss and liver condemnation. Fasciolosis has been recognised by the WHO as a re-emerging neglected tropical disease and it is also of public health interest since it causes human infection as a food-borne parasitic disease; it is estimated that 2.4 million people are infected worldwide in over 70 countries [1].

It is well known that natural hosts do not develop an effective acquired resistance against the infection [2] and that anthelmintic treatment is the best means to control the infection. However, chemical residues in food and their impact on the environment [3, 4], as well as drug resistance reported in various countries [5–8], foster the study of new control methods such as vaccine development, although no vaccine formulation is commercially available to date.

The life-cycle of the parasite inside the animal host is complex: after the infection, the newly excysted juveniles (NEJ) penetrate the intestinal wall within the first two hours post-infection, enter the PC and migrate towards the liver, a process that usually takes about four to six days [9]. By the time *F. hepatica* reaches the mature stage inside its final location in the bile ducts, the disease has become a chronic infection and the immune system of the host has already been affected by the parasite: there is supporting evidence that *F. hepatica* has the capacity to immunomodulate the host’s immune response [10–14].

At the early and late stage of the infection, NEJ and adults worms release a broad variety of antigenic molecules. Some of them include excretory-secretory products which mainly consist of proteins [15], exosome-like vesicles and tegument glycoproteins [16, 17] that may trigger local and systemic immune responses, hence the role of the peritoneal cell population is key for understanding the initial stage of the host-parasite interaction.

In *F. hepatica*-infected sheep at the initial stages of the infection, the peritoneal cavity fluid was recently reported to primarily consist of lymphocytes, macrophages and eosinophils, with lymphocytes and macrophages being the predominant cell populations and displaying a varying ratio along the course of infection [18]. Therefore, these two cell populations are considered as the main phenotypes of interest since they are thought to be one of the first immunocompetent cells involved in the early immune response once NEJ reach the PC. In this regard, it has been shown in a murine model that liver fluke NEJ are killed inside the peritoneal cavity [19], and in a previous study it has been observed that peritoneal macrophages (pMΦ) from rats developed cytotoxic mechanisms against *F. hepatica* NEJ [20]. Recently, it has been confirmed in an *in vivo* model that migrating *F. hepatica* NEJ causes alternative activation of pMΦ [18] and apoptosis in peritoneal leukocytes in sheep [21].

The aim of this study was to determine the immunophenotype of the PCP using flow cytometry to better comprehend its dynamics during the early and late stage of infection in sheep experimentally challenged with *F. hepatica*.

## Methods

### Animals

Thirty-seven female Merino-breed sheep, aged 6 months, were used for the study. Before commencing the study, all animals were confirmed to be free of *F. hepatica* infection by faecal analysis and by an in-house developed ELISA using microplates coated with recombinant *F. hepatica* cathepsin L1 (FhCL1) for detection of specific antibodies. Furthermore, all sheep were given Ivermectin (Noromectin®, Karizoo, Barcelona, Spain) and Diclazuril (Rumicox®, Esteve, Barcelona, Spain) in order to exclude potential presence of parasites. An individual clinical monitoring of the animals was conducted during the trial, which included a weekly clinical examination, and blood sampling for assessment of complete blood counts (data not shown). Simultaneously, faecal samples were taken weekly (from week 0 to week 14 of the trial) for detection of eggs belonging to gastrointestinal worms or *Eimeria* spp. oocysts. In addition, animals were housed in covered pens in clean and healthy conditions in order to avoid the appearance of other pathogens, and were fed daily with hay and commercial pelleted ration.

### Experimental design

The study was designed to allow a comparative analysis of animals between the early and late stage of infection with *F. hepatica*, hence sheep were randomly allocated into three groups according to the slaughtering time. Group 1, 2 and 3 consisted of 20, 10 and 7 animals, respectively. At day 0 of the trial, animals from Group 1 (early stage) and 2 (late stage) were experimentally challenged with a single dose of 150 metacercariae of *F. hepatica* of bovine origin (Ridgeway Research Ltd., St Briavels, UK) administered in gelatine capsules, using a dosing gun. The twenty animals from Group 1 were sacrificed in batches of five sheep at each one of the following time points: 1, 3, 9 and 18 days post-infection (dpi). Animals from Group 2 and 3 were slaughtered 14 weeks post-infection (wpi). Animals from Group 3 were not infected and remained as uninfected control group (UC). All animals were humanly euthanised by an intravenous injection of T61® (Intervet, Barcelona, Spain).

### Parasitological methods: egg output and liver fluke burden

Sheep of the late stage of infection study (Group 2) were sampled for detection of egg output and liver fluke burden. From week six post-infection (wpi) onwards, faecal samples were collected weekly from each animal and faecal examinations were performed using a flotation method for detection of *F. hepatica* eggs. In brief, 3 g of faeces were thoroughly mixed with 42 ml of saturated ZnSO_4_ solution and eggs were counted using a modified McMaster method [22]. Each sample was analysed in duplicate and results were expressed as mean of eggs per gram of faeces.

During necropsy, the gall-bladder and the main bile ducts were opened and the liver was dissected and carefully examined for the presence of liver flukes. The liver was then cut into small pieces and placed into warm water (45 °C) for 30 min to collect remaining flukes which were not observed during bile duct opening. Finally, all liver flukes were counted.

### Isolation of peritoneal cavity cells

A peritoneal lavage was conducted immediately after the slaughtering of each of the animals to obtain the peritoneal fluid. First, the ventral area of the abdomen was shaved and disinfected with polyvinylpyrrolidone iodine 10% (AGB, Madrid, Spain). A small incision (1–2 cm) was made in the skin over the midline and subcutaneous tissue was dissected. The *linea alba* and peritoneum were sectioned with blunt scissors to avoid haemorrhage. A 40-cm-long cannula connected to a syringe was inserted into the abdominal cavity and 60 ml sterile DPBS containing 9500 UI of heparin (warmed to 37 °C; Eurotubo®, Deltalab, Madrid, Spain) was injected into the abdominal cavity. After gently massaging the abdomen for 1 min, 40 ml of peritoneal fluid were withdrawn. Then, peritoneal fluid was centrifuged at 2300× *g* for 5 min and the supernatant was discarded. Cell pellets were resuspended again in DPBS and incubated for 15 min in an erythrolysis buffer (0.15 M NH_4_Cl, 10 mM KHCO_3_, 0.1 mM disodium EDTA, dH_2_O). A second centrifugation step (2300× *g* for 5 min) was performed to eliminate lysed erythrocyte membranes and the pellet was resuspended in 1 ml of medium. After that, the concentration of peritoneal cells was quantified using a Trypan Blue exclusion technique. The final concentration of the cells was then adjusted to 1 × 10^6^ cells/ml in order to carry out a flow cytometry assay.

### Cell population identification and isolation

Different monoclonal antibodies were used to determine the immunophenotype of the peritoneal cavity cell populations in uninfected and infected sheep at 1, 3, 9 and 18 dpi and at 14 wpi by flow cytometry analysis. Main lymphocyte subpopulations were identified separately as TCD4 and TCD8. WC1 was used to select the γδTCR cells. MHC class II was used for identification of APC, and CD14 was used as a marker for macrophages. CD83 was used to identify dendritic cells.

Flow cytometry acquisition was performed with a CyFlow Cube 6® cytometer (4 colours + FSC + SSC; Sysmex-Partec, Barcelona, Spain). Briefly, 200 μl of peritoneal fluid was diluted with 200 μl of phosphate buffered saline (PBS, pH = 7.2) and gently stirred. Afterwards, samples were incubated at 4 °C for 30 min in darkness with the different conjugated antibodies (Table 1) diluted at 1:400, according to the manufacturer’s instructions. All antibodies, with the exception of the mouse anti-human CD83, have been previously used for flow cytometry assay in peripheral blood and lymphoid organs of ovines and goats [23, 24]. As negative control antibodies, mouse isotype control IgG1 and IgG2a [Bio-Rad (formerly AbD-Serotec) Kidlington, UK] were used for FITC and RPE labels, respectively.

**Table 1:** Monoclonal antibodies used in flow cytometry analysis of peritoneal cavity cell populations

Peritoneal cell immunophenotypes	Antibodies	Fluorochromes	Clones	Reference
Isotype controls				
Control FITC	mouse control IgG1	FITC	–	MCA928F
Control RPE	mouse control IgG2a	RPE	–	MCA929PE
Cell populations				
TCD4	mouse anti-sheep CD4	RPE	clone 44.38	MCA2213PE
TCD8	mouse anti-bovine CD8	FITC	clone CC63	MCA837F
WC1^+^γδ	mouse anti-bovine WC1	FITC	clone CC15	MCA838F
CD14	mouse anti-bovine CD14	FITC	clone CC-G33	MCA2678F
CD83	mouse anti-human CD83	FITC	clone HB15e	MCA1582F
MHCII	mouse anti-bovine MHC class II	FITC	clone IL-A21	MCA2445F

Then, the samples were centrifuged at 2300× *g* for 5 min and the supernatant was discarded. After that, two washes with PBS (centrifuged at 2300× *g* for 5 min) were carried out. Finally, cells were resuspended in PBS before acquisition.

Cells were identified by their morphological features gated as defined by their forward and side-scatter profiles (FSC *vs* SSC dot plot) in order to exclude cellular debris. Briefly, cellular debris were excluded by gating the leukocyte populations of interest based on the FSC-SSC (see panel 2.1 in Additional file 1: Figure S1). Once each of the leukocyte populations were defined as mentioned (i.e. lymphocytes, macrophages or dendritic cells), 10,000 events were counted and cell identification was then performed. The immunophenotype of interest was identified using the specific fluorescence channel *vs* SSC dot plots (see panels 3.1 and 3.2 in Additional file 1: Figure S1). For FITC-labelled antibodies, the FL1 channel (green detector, 488 nm laser, 536/40 nm) was used; for CD4-RPE antibody, the FL2 channel (orange detector, 488 nm laser, 590/50 nm) was used. The different peritoneal cell populations from all animals and from each of the slaughtering time points were determined separatedly, in different tubes. Having obtained the number of each immunophenotype, the percentage of the cell subset from the total leukocyte subpopulations was determined (on the basis of 10,000 events).

Results were analysed for changes in fluorescence and expressed as the mean of the percentage of each slaughtering time point. Data was analysed using Infinicyt^TM^ Software v.1.8 (Cytognos, Salamanca, Spain).

### Statistical analysis

Statistical analysis was performed with GraphPad Prism v.6.0 (GraphPad Software, Inc., San Diego, CA, USA). The Kolmogorov-Smirnov test was applied to evaluate if distributions were parametric. Comparison between pairs of groups were made using the two-tailed Mann-Whitney U-test for non-parametric distributions. Data from each stage of the infection (Groups 1 and 2) were statistically compared to data from control animals (Group 3). *P-*values of 0.05 or lower were considered statistically significant.

## Results

### Parasitological results

**Fig. 1:**
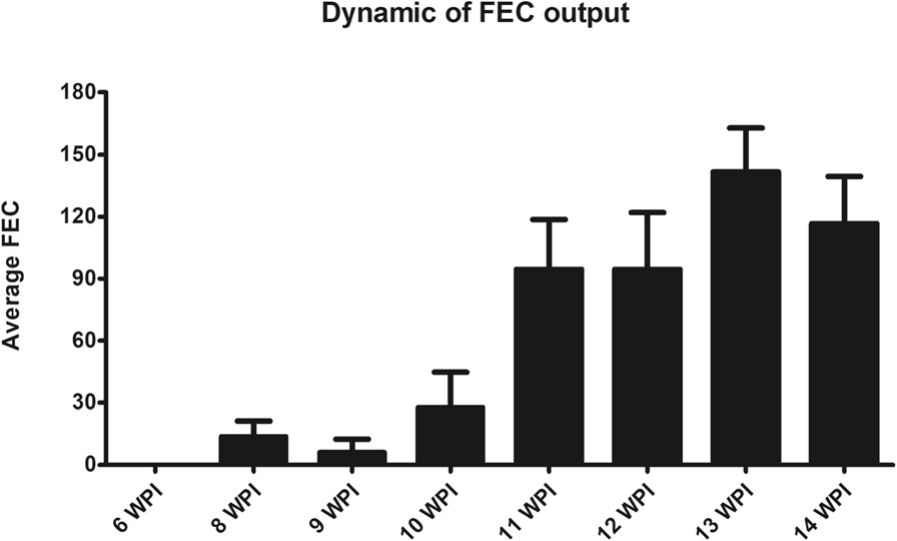
Faecal egg output during the course of infection from animals of Group 2 (*n* = 10). Columns show the mean values of egg counts per week in infected animals from 6 until 14 wpi. Error bars represent standard error

The number of parasites in animals from Group 2 is expressed as mean ± standard deviation (SD). The mean liver fluke burden was 67.78 ± 13.85 which represents an implantation rate of 45.19 ± 9.24%. The dynamics of egg output is shown in Fig. 1. Eggs were first detected at 8 wpi and all animals were positive to egg detection at 11 wpi. The egg output showed a progressive increasing trend from 10 wpi onwards, reaching a maximum level at 13 wpi.

In regard to the clinical monitoring of the animals during the trial, no clinical manifestations of disease, eggs or coccidian oocysts (apart from *F. hepatica* eggs in G2) were detected along the course of the trial.

### Peritoneal leukocyte populations

Results of peritoneal leukocyte populations during the early and late stage of infection are expressed as the percentage (mean ± range) of each cell subpopulation (i.e. for TCD4, TCD8 and WC1^+^γδ results are expressed as the percentage of these three cell subsets of the total lymphocytes). The APC are represented as the percentage of MHCII. For macrophages and dendritic cells, CD14 and CD83, respectively, were used. Values are shown in Table 2.

**Table 2:** Percentage of peritoneal cells

Cell populations	Percentage (%)					
	Group 1				Group 2	Group 3
	1 dpi (*n* = 5)	3 dpi (*n* = 5)	9 dpi (*n* = 5)	18 dpi (*n* = 5)	14 wpi (*n* = 10)	NegCtl (*n* = 7)
TCD4	12.42 (8.14–18.29)	27.24 (19.57–33.73)	37.00 (34.18–40.11)	18.68 (14.00–23.45)	33.68 (25.08–45.21)	30.98 (22.09–38.56)
TCD8	13.47 (10.13–15.65)	25.04 (18.98–28.92)	9.24 (7.52–12.42)	22.86 (17.73–34.22)	29.93 (13.25–41.55)	18.40 (2.59–22.91)
WC1^+^γδ	5.28(4.30–6.40)	1.71 (1.13–2.44)	5.22 (3.40–7.69)	7.41 (4.85–10.93)	7.30 (4.57– 10.33)	2.28 (1.13–2.44)
CD14	60.15 (39.82–78.17)	63.48 (49.86–79.07)	41.42 (38.76–43.31)	31.84 (15.50–62.19)	19.18 (11.19–29.85)	69.44 (53.68–86.9)
CD83	22.78 (8.58–47.50)	2.17 (0.52–7.70)	11.89 (9.48–14.06)	23.21 (18.16–33.71)	6.17 (1.62– 8.00)	23.21 (20.75–27.43)
MHCII	48.68 (27.55–63.02)	9.04 (1.84–16.53)	22.54 (20.91–24.62)	40.23 (14.98–93.13)	6.66 (3.44–14.59)	62.00 (65.14–84.24)
Total cell count (40 ml)	598.16 × 10^6^	298.32 × 10^6^	3771.60 × 10^6^	19,918.00 × 10^6^	1002.40 × 10^6^	418.20 × 10^6^
Concentration (cells/ml)	14.95 × 10^6^	7.46 × 10^6^	94.29 × 10^6^	497.95 × 10^6^	25.06 × 10^6^	10.46 × 10^6^

Cell immunonophenotypes are expressed as the mean of the percentage of each time point of challenged sheep from Group 1 (early stage) and from Group 2 (late stage) and UC animals (Neg. Ctl). The range of each immunophenotype are shown in parentheses. Total peritoneal cell counts and concentration of cells are expressed as the mean of each Group at the different time points

### Early stage of the infection

**Fig. 2:**
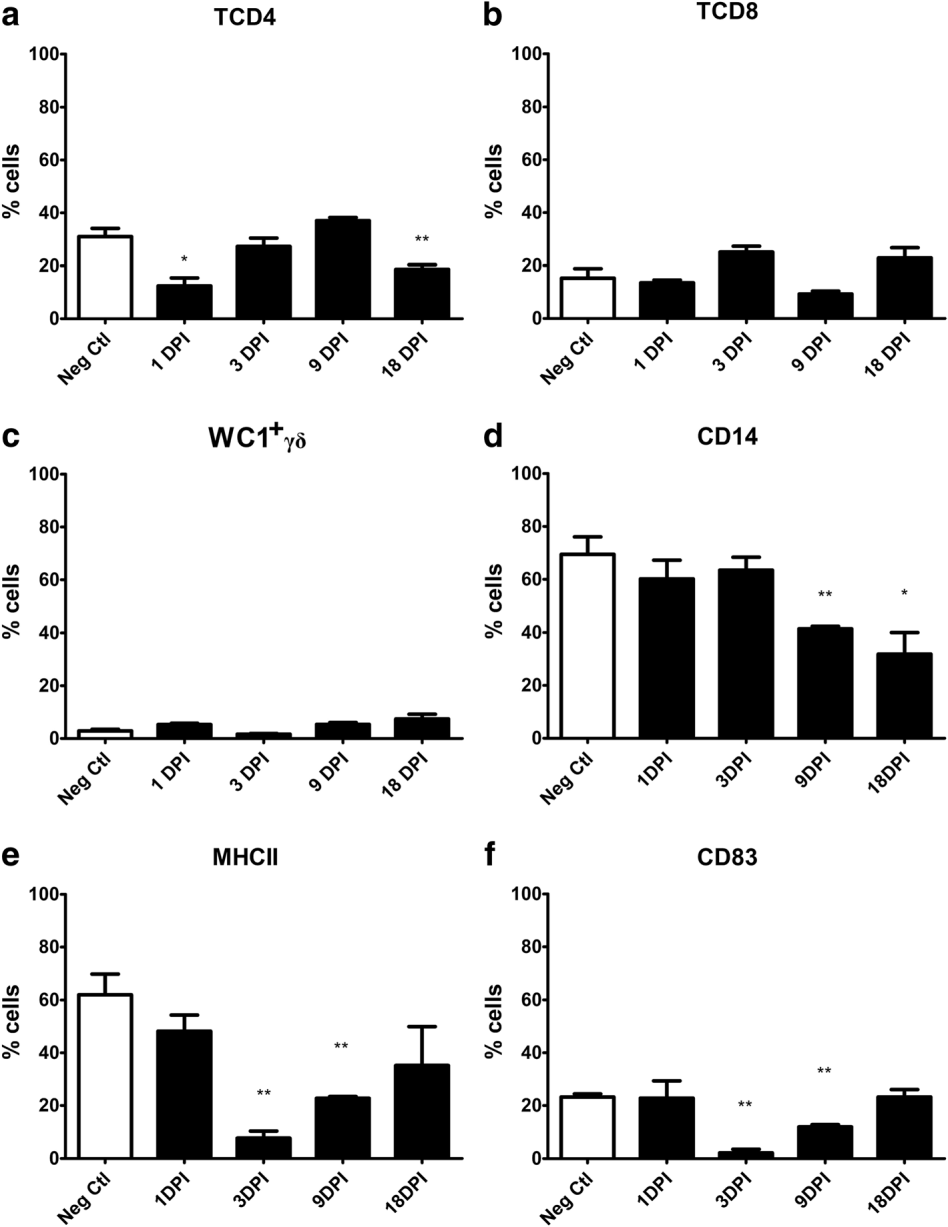
Percentage of cell populations in the peritoneal fluid during the early stages of infection. The specific cell populations are shown as follows: (**a**) TCD4; (**b**) TCD8; (**c**) WC1^+^γδ; (**d**) CD14; (**e**) MHCII; (**f**) CD83. Cell subsets of uninfected (Neg. Ctl) and infected sheep (Group 1) are shown in columns. Mann-Whitney U-test was used to compare data from Groups 1 and 3. Values represent the mean ± standard deviation (SD). Asterisks indicate different levels of significance between groups: *P ≤ 0.05, **P ≤ 0.01

The total number of cells obtained from 40 ml of peritoneal fluid at 1, 3, 9 and 18 dpi was 598.16 × 10^6^, 298.32 × 10^6^, 3771.60 × 10^6^ and 19,918.00 × 10^6^, respectively (Table 2). The dynamics of the percentage of the peritoneal lymphocytes and APC in the negative and infected animals during the early stage of infection is shown in Fig. 2a-f. In the infected animals, flow cytometry analyses showed that lymphocyte cell subpopulation dynamics was different for TCD4, TCD8 and WC1^+^γδ cells. No significant differences with respect to the UC Group were detected for TCD8 and WC1^+^γδ cells, although slight non-significant variations were observed at different time points. The dynamics of TCD4 cells was irregular, with an initial decrease at 1 dpi (*U* = 0, *df* = 8, *P* = 0.0357), then an increase at 3 and 9 dpi and again a significant decrease at 18 dpi (*U* = 1, *df* = 8, *P* = 0.0159) when compared to animals of the UC.

Regarding the dynamics of APC, a different tendency was observed during the early stage of infection for CD14, MHCII and CD83 cells (Fig. 2d-f). CD14 cells exhibited a decreasing trend, with a significant decrease at 9 (*U* = 0, *df* = 8, *P* = 0.0079) and at 18 dpi (*U* = 2, *df* = 8, *P* = 0.0317) when compared to the UC. The dynamics of MHCII and CD83 cells was quite similar with no significant modifications at 1 dpi and a significant decrease at 3 and 9 dpi with respect to the UC sheep. The decrease of MHCII cells was statistically significant at 3 dpi (*U* = 0, *df* = 8, *P* = 0.0079) and 9 dpi (*U* = 0, *df* = 8, *P* = 0.0079); CD83 cells were significantly reduced at 3 and 9 dpi (*U* = 0, *df* = 8, *P* = 0.0079) with respect to the uninfected sheep. When compared, both MHCII and CD83 cells showed the same increasing pattern from 3 to 18 dpi.

### Late stage of the infection

The total number of cells obtained from animals of the Group 2 and 3 was 1002.40 × 10^6^ and 418.20 × 10^6^, respectively (Table 2).

**Fig. 3:**
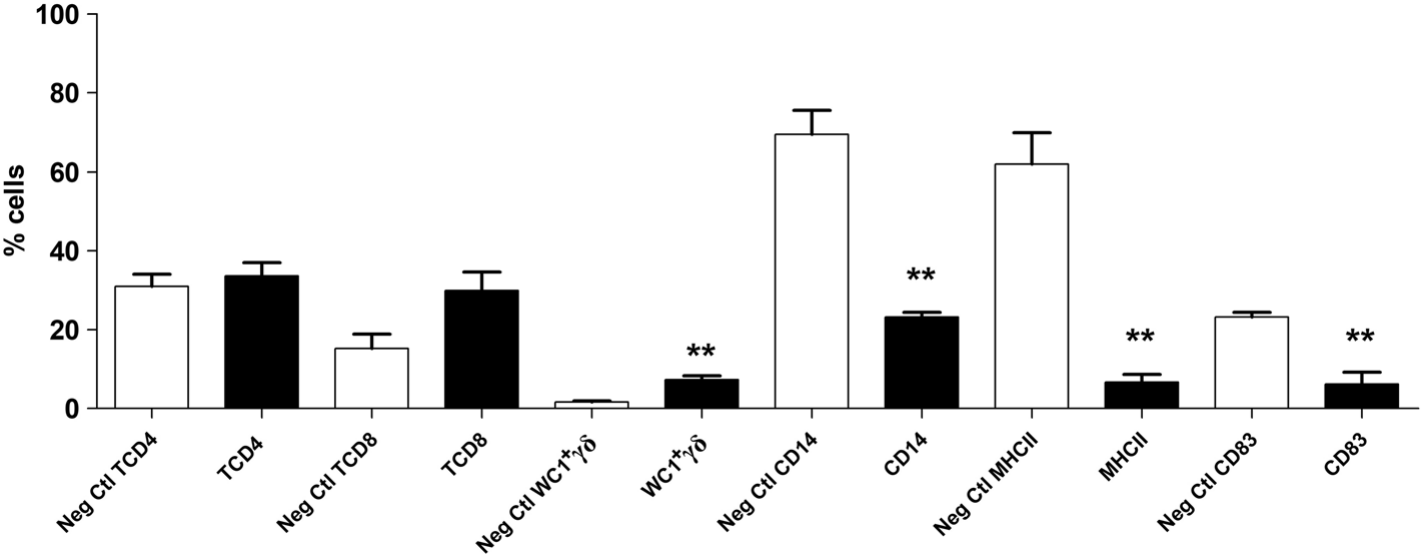
Percentage of peritoneal cell populations during the late stages of infection. Each cell subset of uninfected (Neg. Ctl) and challenged animals (Group 2) are shown in columns. Mann-Whitney U-test was used to compare data from Groups 2 and 3. Values represent the mean ± standard deviation (SD). Asterisks indicate different levels of significance between groups: *P ≤ 0.05, **P ≤ 0.01

At 14 wpi, the percentage of both TCD4 and TCD8 cells showed no significant differences compared to the uninfected group, although a slight but statistically significant higher level of WC1^+^γδ cells (*U* = 0, *df* = 15, *P* = 0.0079) was detected in the infected sheep (Fig. 3).

A noticeably lower percentage of CD14, MHCII and CD83 cells in infected animals was detected when compared to the uninfected sheep. Statistical analysis showed a significant decrease for the three cell populations (*U* = 0, *df* = 15, *P* = 0.0079).

## Discussion

Peritoneal leukocyte population in sheep includes mainly lymphocytes and macrophages (and DC), with a minor proportion of eosinophils and neutrophils [21]. Within these cells, macrophages and DC are considered APC which play a crucial role in the innate and local immune response. Meanwhile, T lymphocytes are known to be of key importance as they immunoregulate the host immune response through various mechanisms such as cytokine production. Currently, many *F. hepatica* molecules have been shown to produce severe effects on these cell populations [13, 25–30], hence many studies are focusing on these particular cell populations. The host immune response against *F. hepatica* is believed to be more effective during the initial peritoneal or early hepatic migratory stages [20]. Therefore, we aimed to (i) determine the immunophenotype and the dynamics of the PCP, which are believed to be one of the main immunocompetent cells involved in the early immune response once NEJ have entered the PC, and to (ii) carry out a comparative study between the acute and chronic stage of the infection.

In our study we observed that the dynamics of the lymphocyte subsets did not show major variations in the peritoneal cavity during the early and late stage of the infection. Peritoneal macrophages showed a decreasing trend whereas MHCII and CD83 cells tended to develop an increasing trend along the infection. Indeed, at the beginning of the infection, when NEJ are migrating through the peritoneal cavity (3–9 dpi), the MHCII and CD83 cells were significantly reduced; later on, at 9–18 dpi, when the peritoneal migratory stage of the parasite is supposed to have finished, there was a significant reduction of CD14 cells. Subsequently, all three populations were again significantly reduced in the late stage of the infection in comparison to the UC.

TCD4 cells were the more prominent subpopulation both at the early and late stage of the infection and it was the only one that was modified during the early stage, with a significant decrease at 1 and 18 dpi. TCD8 cells, the second population, did not vary significantly along the infection, but WC1^+^γδ cells, the minority subpopulation, was slightly increased in the chronic stage. To our knowledge, there are no previous *in vivo* studies in *F. hepatica*-infected ruminants regarding the dynamics of peritoneal lymphocyte phenotypes during the early and late stage of the infection. In *F. hepatica*-infected goats, our group previously studied the dynamics of circulating TCD4, TCD8 and WC1^+^γδ cells from peripheral blood during chronic stages of infection, and a significant reduction of TCD4 cells was found at 5 and 12 wpi in respect to the UC, whereas no significant differences were noted for TCD8 and WC1^+^γδ cells [31]. The different dynamics of TCD4 and WC1^+^γδ cells in chronic stages of the present study compared to the previous study in goats suggests a different behaviour of peritoneal and peripheral TCD4 and WC1^+^γδ cell subsets. Alternatively, this may be due to different cell subset recruitment in *F. hepatica*-infected sheep and goats. Recently it was reported that total peritoneal lymphocyte population was only increased at 9 dpi in *F. hepatica*-infected sheep in respect to UC, and a decrease was observed at 18 dpi, probably due to the increment in the recruitment of eosinophils [18]. In this regard, in some animal models experimentally infected with enteric helminths, there is supporting evidence that once infection was overcome, a persistent TCD4 population, in a significantly high frequency, occurs at the peritoneal cavity [32], something we partly saw in our study at the chronic stage, when all liver flukes were thought to be already established in the liver. Due to their influence on the polarisation of the immune response in subsequent reinfections, TCD4 cells have been described as the major population for the development of resistance to helminth infection, as occurs with gastrointestinal nematodes [33, 34]. In addition, this cell population is also known to control immunoregulatory mechanisms during helminth infections by means of cytokine secretion [28].

An unexpected result of our study was the observation of a lack of significant increase in the peritoneal T cell population during the early stages, as it has been described in *F. hepatica*-infected rats, in which peritoneal TCD4 and TCD8 were significantly augmented at early stages [28]. In fact, the recruitment of these T cell subpopulations (TCD4, TCD8 and WC1^+^γδ) in the organ in which the infection was established has been reported for ruminants in helminth infections [35]. In infected goats, we previously detected that the early hepatic lesions due to the penetration of the NEJ in the liver occurred in between 7 and 9 dpi, with a noticeable cellular infiltrate surrounding the initial hepatic lesions, mainly composed of TCD4 and TCD8 [31]. Therefore, it may be hypothesised that as *F. hepatica* NEJ migration through the PC only occurs during the first 4–6 dpi [9], this period might be insufficient to develop a significant increment in the recruitment of peritoneal T cells, which might not affect the recruitment of the lymphocyte subsets in the liver during the early stage.

With respect to the WC1^+^γδ T cells, an increase in the percentage was only detected at the late stage of the infection. The influence of the variation of the dynamics of WC1^+^γδ observed on the immune response during the early and late stage of the infection still remains to be elucidated. There is supporting evidence that γδ T cells may play an important role in the immune response as they can mediate effector activities such as production of INF-ɣ or TNF-α [36]. Moreover, in cattle the WC1^+^γδ T cells were shown to act as APC for αβ T cells [37]. This might condition some immunoregulatory processess which can have an effect on the outcome of the infection in reinfected animals, since this cell population is known to develop memory activity against different pathogens [38, 39].

One of the most noteworthy findings of our study was the influence of the infection on peritoneal CD14, CD83 and MHCII cells over time. A significant decrease could be observed in the early stage in the three studied subpopulations: during the period of peritoneal migration of juvenile flukes (3–9 dpi) for CD83 and MHCII cell populations, and at the time of penetration in the liver parenchyma for the CD14 cell population. Similarly, a marked and significant reduction of the three subpopulations was demonstrated at the chronic stage when compared to UC. Although there are very few studies focused on the dynamics of the peritoneal macrophages (CD14), dendritic cells (CD83) and MHCII cells during infection in ruminants, and the results of these studies are somehow dissimilar [18, 21], there is strong evidence of the modulation of APC functions by *F. hepatica* antigens. In previous *in vitro* studies conducted in mice, tegumental antigens were shown to suppress DC maturation and function [27, 30], *F. hepatica* glycans showed to downregulate the expression of MHCII [25] and some other ES-derived proteins and peptides modulated DC activity by different mechanisms [14, 40]. We have also demonstrated that *F. hepatica* also modulates the oxidative response of pMΦ, inducing an increase in nitric oxide and hydrogen peroxide production during the early stage of infection in sheep [41]. Moreover, we previously described a specific induction of apoptosis in peritoneal leukocyte populations in the early stage of *F. hepatica* infection in sheep [21], which probably affects the subpopulations of CD14, MHCII and CD83 analysed in this study.

In the chronic stage of the infection, the percentages of the three APC subpopulations were also significantly reduced in comparison to UC. At this time, adult parasites have been located in the bile ducts for several weeks and the activity of some peritoneal macrophages and dendritic cells remains uncertain. It has been suggested that the peritoneum acts as an important lymphoid organ where presentation of antigen to the immune cells takes place and is followed by their migration to the inflammatory site [35] and the role of some particular peritoneal APC could be related to the recruitment of macrophages and lymphocytes in the inflammatory infiltrate surrounding the large bile ducts [21]. However, recent transcriptomic analysis in *F. hepatica*-infected sheep have revealed a complex and different pattern of response in early [42] and late stages of infection [43] and the recruitment and functionality of different cell populations can be modulated in different ways in the different tissues of the host [44].

In summary, we have analysed the cellular immune response elicited in the peritoneal cavity in sheep infected with *F.* hepatica and provided novel data regarding immune cell recruitment over the course of the infection. To our knowledge, this is the first report of the use of flow cytometry for the assessment of the dynamics of local immunocompetent cells at the abdominal cavity in the *F. hepatica* natural host.

## Conclusions

We have identified the immunophenotype and the recruitment of the peritoneal cell population in sheep uninfected and infected with *F. hepatica* during the early and late stage of the infection. There is no statistically significant increment in the recruitment of the peritoneal TCD4, TCD8 and WC1^+^γδ cells along the course of infection, with the exception of WC1^+^γδ cells at the chronic stage. The dynamics of the CD14 cells recruitment at the peritoneal cavity displayed a decreasing trend during the early stage and was significantly decreased at the chronic stage of the infection. CD83 and MHCII cells developed an increasing trend during the early stage of infection, and were significantly decreased at the chronic stage of the infection.

## Additional files

Additional file 1: **Figure S1.** Gating strategy for the identification of cell immunophenotypes by flow cytometric analysis. **1.** Dot-plot samples (1): FSC and SSC in log scale are faced in order to exclude debris. Red square shows the leukocyte populations of interest. **2.** Dot-plot (2.1) and histogram (2.2): once leukocyte populations are gatted, lineal FSC is faced to log SSC so that white cells can be shown and identified properly. The histogram is an extra support for the correct gating in leukocyte subsets. **3.** Dot-plots for fluorochromes (3.1 and 3.2): according to the fluorochrome in each antibody, RPE or FITC channels are faced to log SSC, and in each dot-plot it is only shown the subset of interest which was gated in dot-plot 2. (DOCX 378 kb)

## Abbreviations

APC: Antigen presenting cells
DC: Dendritic cells
dpi: Days post-infection
NEJ: Newly excysted juveniles
PC: Peritoneal cavity
PCP: Peritoneal cell populations
pMΦ: Peritoneal macrophages
UC: Uninfected control
WHO: World Health Organization
wpi: Weeks post-infection
