# Supplementary material for: Comparative dynamics of peritoneal cell immunophenotypes in sheep during the early and late stages of the infection with Fasciola hepatica by flow cytometric analysis
Source: Parasit Vectors. 2018 Dec 14;11:640. doi: 10.1186/s13071-018-3250-5 (PMC6295066; doi:10.1186/s13071-018-3250-5)
Supplement: Supplementary file 1 — Figure S1. Gating strategy for the identification of cell immunophenotypes by flow cytometric analysis. 1. Dot-plot samples (1): FSC and SSC in log scale are faced in order to exclude debris. Red square shows the leukocyte populations of interest. 2. Dot-plot (2.1) and histogram (2.2): once leukocyte populations are gatted, lineal FSC is faced to log SSC so that white cells can be shown and identified properly. The histogram is an extra support for the correct gating in leukocyte subsets. 3. Dot-plots for fluorochromes (3.1 and 3.2): according to the fluorochrome in each antibody, RPE or FITC channels are faced to log SSC, and in each dot-plot it is only shown the subset of interest which was gated in dot-plot 2. (DOCX 378 kb) [file 13071_2018_3250_MOESM1_ESM.docx]

**Additional file 1: Figure S1.** Gating strategy for the identification of cell immunophenotypes by flow cytometric analysis

**
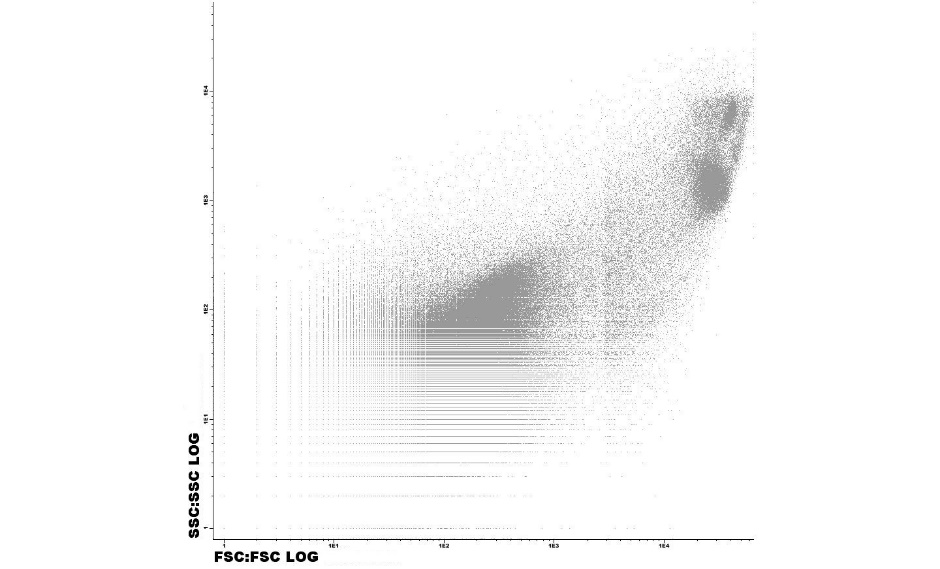
**

**1**


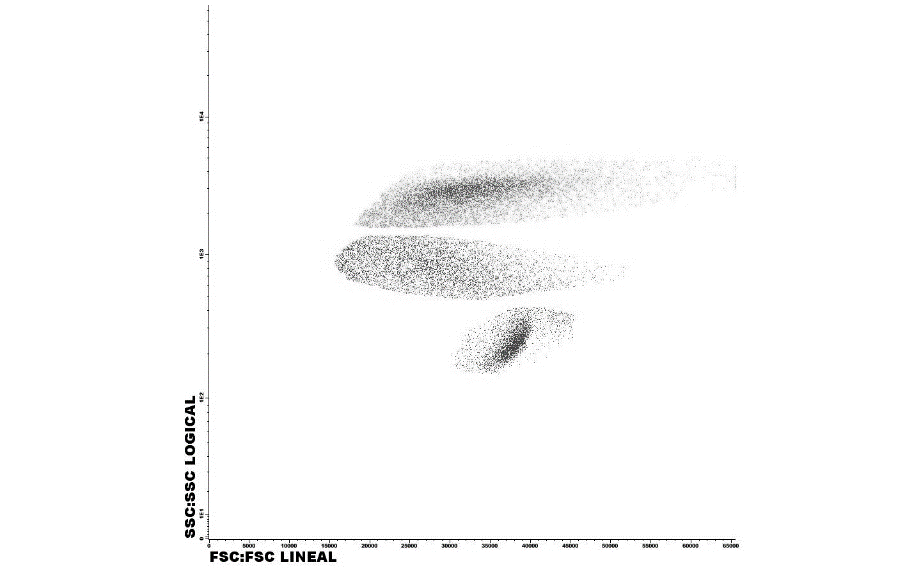

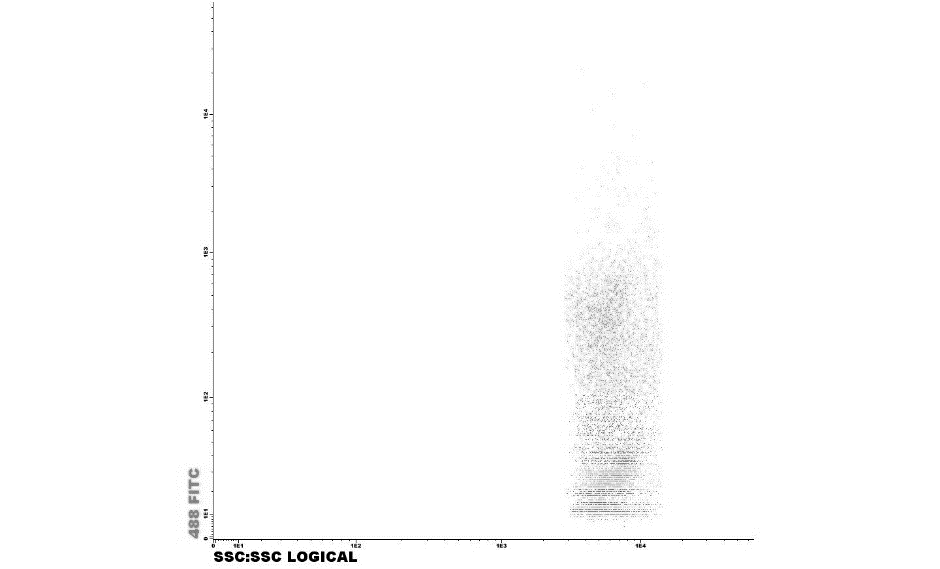


**2.1**

**3.2**


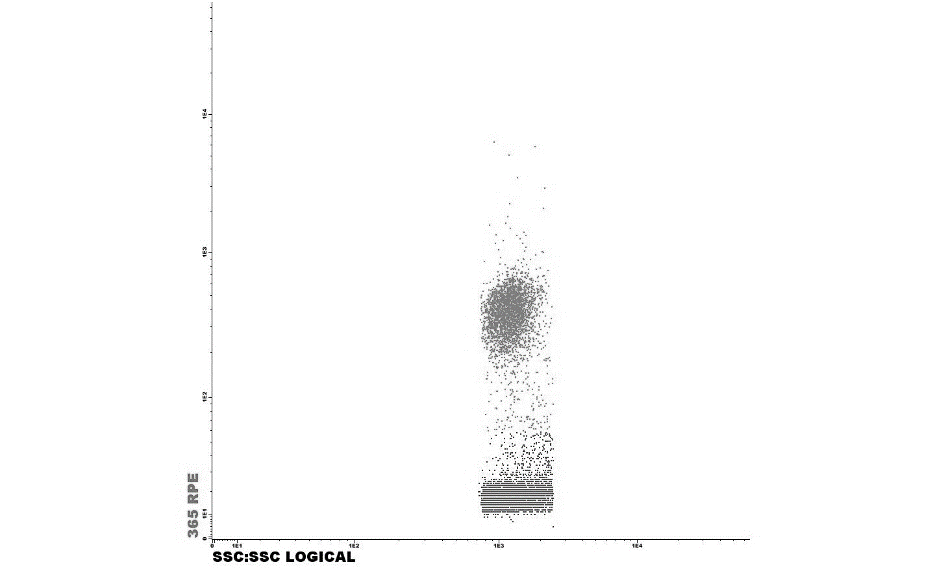


**3.1**


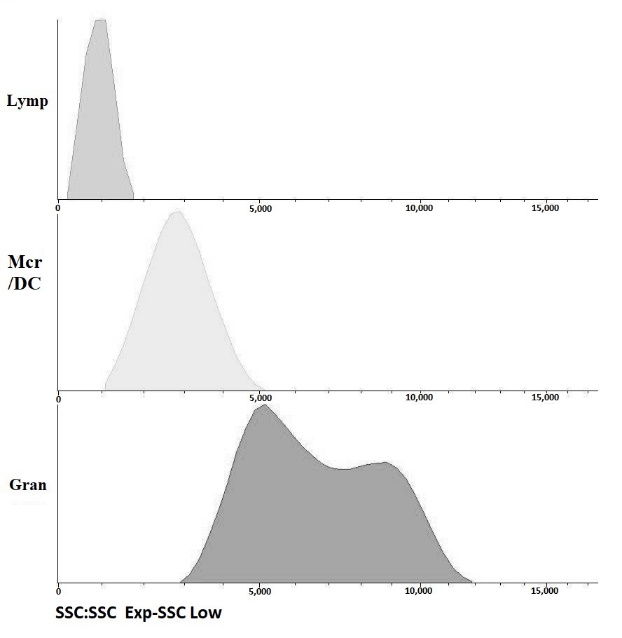


**2.2**

1. **Dot-plot samples (1):** FSC and SSC in log scale are faced in order to exclude debris. Red square shows the leukocyte populations of interest.
2. **Dot-plot (2.1) and histogram (2.2):** once leukocyte populations are gatted, lineal FSC is faced to log SSC so that white cells can be shown and identified properly. The histogram is an extra support for the correct gating in leukocyte subsets.
3. **Dot-plots for fluorochromes (3.1 and 3.2):** according to the fluorochrome in each antibody, RPE or FITC channels are faced to log SSC, and in each dot-plot it is only shown the subset of interest which was gated in dot-plot 2.
